# Objective Quantum Fields, Retrocausality and Ontology

**DOI:** 10.3390/e23060749

**Published:** 2021-06-14

**Authors:** Peter D. Drummond, Margaret D. Reid

**Affiliations:** Department of Physics and Astronomy, Swinburne University of Technology, Melbourne 3122, Australia

**Keywords:** quantum, ontology, reality, measurement, fields, retrocausality

## Abstract

We compare different approaches to quantum ontology. In particular, we discuss an interpretation of quantum mechanics that we call objective quantum field theory (OQFT), which involves retrocausal fields. Here, objective implies the existence of fields independent of an observer, but not that the results of conjugate measurements are predetermined: the theory is contextual. The ideas and analyses of Einstein and Bohr through to more recent approaches to objective realism are discussed. We briefly describe measurement induced projections, the guided wave interpretation, many-universes, consistent histories and modal theories. These earlier interpretations are compared with OQFT. We argue that this approach is compatible both with Bohr’s quantum complementarity and Einstein’s objective realism.

## 1. Introduction

There are few issues in science more important than the problem of “what is”. This question is at the heart of philosophy since Plato’s cave analogy [1], and it has led to scientific breakthroughs, like Rutherford’s discovery of the nucleus [2]. Such questions have been common in quantum mechanics since the early discussions of Bohr [3], Einstein [4] and Schrödinger [5]. The fundamental issue is understanding the nature of reality.

Today, there are many viewpoints on this question among physicists. Recent analyses present the case that the wave-function has a real existence [6]. There is also the notion that it simply carries information [7]. An old argument against wave-function or “quantum state” reality is that the Copenhagen approach implies that the wave-function “collapses” on measurement [8]. This has been thought to imply a lack of wave-function reality.

Here, we explore another viewpoint, based on time-symmetry, and give arguments in favour of it. A quantum model has been proposed in [9], in which there are objective fields with a probability based on the Q-function of quantum mechanics [10]. In this approach, the quantum state is a computational aid, which one may wish to use, or not. The fields are defined to exist in a Lorenz-invariant space-time manifold, and their probability is determined by a probabilistic action principle without requiring imaginary time [11]. The important feature is that the theory is time-symmetric and involves retrocausality. This may lead to a resolution of macroscopic paradoxes [9,12], and gives a way to address the measurement problem.

One can also ask “why even worry about ’what is’?”. This is much easier to explain. Physicists have always asked about deeper realities. This has been highly productive, whether investigating what lies inside the atom, or outside the solar system. Max Born asked in his Nobel lecture about quantum mechanics, “what is the reality which our theory has been invented to describe?” [13]. In this case, the question is: what, if any, objective reality lies beneath the abstract formalism of quantum mechanics?

The argument against the wave-function being a complete description of reality is straightforward. Measurement is ill-defined, in the sense that whatever a measurement does is no different in principle to any other interaction. There is no “collapse” in normal quantum time-evolution. The idea that collapse occurs on measurement is often taken to imply that the wave-function is not objectively real. However, it could describe a state of knowledge that changes on measurement. This does not imply anything strange, just acquisition of knowledge.

If a retrocausal approach seems too simple, we might answer: why not? An objective model of fields can be relativistic. Retrocausal dynamics has been studied in physics many times since the work of Dirac, Feynman and Wheeler on electrodynamics [14,15]. It has also been used to understand Bell violations [16,17]. It could be that retrocausality also explains quantum measurement randomness. This appears consistent with the vacuum noise present in OQFT.

Many no-go theorems in physics are based inherently on the assumption that the present affects the future, but they disallow the reverse, which often means that they implicitly assume non-contextuality [18]. Other no-go theorems [19,20] have ruled out retrocausal explanations of Bell non-locality, or point to fine-tuning problems for classical noncyclic causal processes [21,22]. None of these rule out an objective, contextual theory with cyclic loops [23], as proposed here. As classical causal relations are negated by quantum states, more general objective theories are required.

A retrocausal approach has the virtue of being conceptually simple compared to some alternatives, like many-worlds theories [24]. It has the great advantage that measurement is treated on the same basis as other processes. There is no special treatment of measurement, with a non-unitary collapse. Measurements involve the same physical laws as anything else. Another advantage is to give a description consistent with relativity, that does not require violation of special relativity with superluminal disturbances, as in early guiding-wave theories [25]. Scientific theories are always provisional, so this proposal of an alternative is an existence theorem, demonstrating that modeling quantum reality with physical field configurations in space-time is not impossible.

The purpose of this paper, in summary, is to discuss an interpretation of quantum mechanics as describing reality via a model of objective quantum fields. For simplicity, we do not treat objective collapse theories [26,27]. These inherently lead to a different set of predictions from quantum mechanics. Such theories also include an asymmetry under time-reversal, and are different to the approach that we outline here. Our focus in this paper is mostly on concepts. We hope that this may be useful for those wishing to obtain a general picture of the philosophy behind the approach, without mathematical details that are derived elsewhere [9,11,28].

## 2. Objective Fields

We now explain how the proposed interpretation of quantum mechanics, objective quantum field theory (OQFT), unifies most of the views of Einstein and Bohr. Our proposal is presented in [9], and is motivated by the Q-function phase space representation. As such, it is an objective model of fields in space and time. It represents the known physics of quantum fields, our most fundamental theory. This approach is compatible with the standard model, is consistent, and empirically viable. It includes random vacuum noise, in accord with experiments that have random results. The observation of eigenvalues, and the violation of Bell inequalities can both be replicated [9,28].

However, the theory must necessarily include a physical model of the measuring instrument to be a complete picture of measurement. This provides a natural, operational way to explain contextual results. The model is different to the earlier interpretations described below. It proposes that there are many potential universes, but not that they all exist [24]. One can imagine having six possible outcomes from throwing a dice. However, they do not all exist simultaneously.

Nor does decoherence play a fundamental role. In OQFT, what is most important for measurement is amplification [12]. This makes microscopic quantum events large enough for our macroscopic neurons to register them. Decoherence occurs in as much as macroscopic signals couple strongly to their environment. This is a consequence, not a cause of measurement.

The model is described by a probability at time *t*, P(ϕ,t), for a set of fields $\phi \left(r\right)$ that are objectively real. These exist in a four-dimensional space-time with a coordinate $r=\left(t,r\right)$. This includes the fact that there are matter-waves. The fields can diffract and interfere, and have quantum statistics implicitly. There are no point-like particles, although fields may be localized due to interactions. This is the difference between using fields, and the particle ontology of Bohm [25]. In the OQFT approach, one defines a vector field amplitude ϕ, and a coherent state projector $\hat{\Lambda }\left(\phi \right)$. The probability of ϕ for a quantum density matrix $\hat{\rho }\left(t\right)$ is a generalization of the Husimi Q-function [10], defined as

(1) $$P\left(\phi ,t\right)=Tr(\hat{\Lambda }\left(\phi \right)\hat{\rho }\left(t\right)),$$

where

(2) $$\int P\left(\phi ,t\right)d\phi =1.$$

Here, P(ϕ,t) is the marginal probability at time *t*, relative to $P\left[\phi \right]$, which is the full space-time functional probability. The generalized phase-space coordinate ϕ includes both Bose and Fermi fields, and $\hat{\Lambda }\left(\phi \right)$ is an outer product of bosonic and fermionic projectors. For example, in the case of a complex scalar bosonic field, as in the Higgs–Englert–Guralnik model [29,30,31], the objective quantum field for *M* discrete modes in a volume *V* is a complex field representing an operator field $\hat{\phi }$:

(3) $$\phi \left(r\right)=\sum_{n}\frac{1}{\sqrt{2E_{n}V}}\left[e^{ik_{n}\cdot r}\alpha_{n}\left(t\right)+e^{-ik_{n}\cdot r}\beta_{n}^{*}\left(t\right)\right].$$

The $k_{n}$ are field momenta, α and β are coherent state vectors for particles $\left(p\right)$ and antiparticles $\left(a\right)$, and the projector ${\hat{\Lambda }}_{b}\left(\phi \right)$ for bosonic coherent states ${|\alpha \rangle }_{p},{|\beta \rangle }_{a}$ is

(4) $${\hat{\Lambda }}_{b}\left(\phi \right)=\frac{1}{\pi^{2M}}|\alpha \rangle {\langle \alpha |}_{p}|\beta \rangle {\langle \beta |}_{a}.$$

As usual, one can take infinite mode limits as M,V→∞, provided the Hamiltonian is renormalisable. Fermionic coherent states require a generalized Gaussian projection operator ${\hat{\Lambda }}_{f}$, which is a function of an antisymmetric real matrix that describes Fermi field correlations [32,33,34,35]. The matrix, after Fourier transforming, represents a product of two operator Majorana fields, $i{\hat{M}}_{i}\left(r_{1}\right){\hat{M}}_{j}\left(r_{2}\right)$.

Objective fields have the hallmark of elements of reality. There is a positive probability density of fields ϕ occurring at a given time, and the total probability is unity. Thus, normal laws of probability hold. Therefore, it is not unreasonable to claim that ϕ exists ontologically, although P(ϕ,t) is epistemic, or knowledge-based. As *P* is equivalent to a wave-function, this implies that the wave-function is also epistemic. It describes knowledge via probability, and therefore is *not* ontological. This is not inconsistent with the phase-space coordinate ϕ being viewed as real.

The task of a physicist is to determine the probabilities that certain trajectories exist. This creates an epistemic distribution of trajectories equivalent to a wave-function. Despite this, a measurement does not change the universe’s trajectory except according to the fundamental action principle. There is a self-consistency requirement, which is not surprising. Cognition has no effect apart from biochemistry, unless it results in the observer’s actions, but this too is part of the universe.

There is an increase of knowledge on measurement, if one wishes to take cognition into account. The information itself exists relative to an observer, their observations, and their deductions. The objective universe is not greatly changed when an observer determines what they know about their apparatus. There are only neurological changes, described by the same laws of physics as everything else. The universe would evolve in a similar way if there were no observers. The moon exists even when unobserved.

More details are given elsewhere [9,11,28], although this is still very much work in progress. Not all the calculations of quantum field theory have been explicitly repeated. Certainly, much remains to be calculated. Conventional quantum mechanics, following Dirac’s description of the Copenhagen approach, is a highly successful theory, with over a century of complex theoretical work behind it. The approach described here is not intended to replace this work. However, it may make computations easier in future, as new techniques are developed.

In the calculations carried out so far [9,28], no inconsistencies with standard QFT were found *provided a model of the measuring instrument is included*. In other words, OQFT gives empirical results similar to those obtained from standard QFT, if one only takes observational account of macroscopic outputs of measuring instruments. This general approach was emphasized in Bohr’s early writings [36,37]. State preparation has rather similar properties. That is, a realistic model of quantum state preparation would require essentially a time-reversal of the measurement argument, with inputs from some operational, macroscopic device. Neither case requires the field coordinate ϕ to have a one-to-one correspondence with a traditional quantum state $|\psi \rangle$.

Detailed results were given for the measurement dynamics of the phase coordinates *x* and *p*, for a single mode field prepared in an eigenstate $|x_{i}\rangle$ of the *X* field quadrature [9,28]. As is well known, the Husimi Q function for an eigenstate $|x_{i}\rangle$ is a Gaussian with a variance (noise η) of order *ℏ* in the phase space variable *x*. Noise is present, even though this is an eigenstate. Solving for the dynamics of the measurement modelled as a parametric amplifier reveals retrocausal trajectories for *x*. The result is that the measurement gives a distinct amplified outcome $gx_{i}$, where *g* is the amplifier gain, with minimal noise to signal ratio. This allows the eigenvalue $x_{i}$ to be inferred from the macroscopic signal, since the noise η is *not* amplified.

Other analyses have recently obtained similar conclusions [38]. Therefore, measurements will lead to a well-defined spectrum of outcomes [28]. In this model, measurement is the process that gives a distinct eigenvalue. Including a measuring instrument is the natural source of contextual measurement results. This is important and was an issue also emphasized very strongly by Bohr. With an appropriate model of an amplifier present, there is no need to carry out projections. In fact, these are not meaningful in an objective model. This approach also provides a source of the random noise observed in measurements, an issue often neglected in realistic models.

We note that the OQFT model is compatible with macroscopic realism, if that concept is carefully defined. This is because a system in a superposition of macroscopically distinguishable eigenstates $|x_{1}\rangle$ and $|x_{2}\rangle$ may be interpreted to be probabilistically in an ‘ontological state’ that will give (after amplification due to the measurement) a definite value, $x_{1}$ or $x_{2}$, for the result of the measurement *X* [9,28]. There is thus a consistency with macroscopic realism, without any need to introduce decoherence. The apparently contradictory claims in the literature of violations of macroscopic Bell inequalities [39] and Leggett–Garg’s macro-realism [40,41] can be explained, once one accounts for the dynamical nature of the measurement process [42]. This does not imply that the system in the superposition was actually in one or the other eigenstate $|x_{1}\rangle$ or $|x_{2}\rangle$ (or indeed in *any* quantum state), prior to measurement. Such an implication would be a much stronger definition of macroscopic realism, which is known to be falsifiable since macroscopic superpositions and mixtures can be experimentally distinguished, although with increasing difficulty in the macroscopic limit [43].

The distinct values of ϕ we use do not correspond to orthogonal projections. The projectors are determined by the coherent states, i.e., the symmetric spaces of Lie groups [44,45,46,47,48,49] corresponding to the field commutators. This is a mathematical criterion, unrelated to any measurement. A phase-space coordinate ϕ evolves in time to give a phase-space trajectory ϕ(t). The trajectory ϕ(t) is a set of classical fields in space-time, which exists at all space-time events. For bosons it can be written as $\phi \left(t,x\right)$. In the case of fermions, one has to include field correlations.

## 3. Causality and Retrocausality

The idea of future boundary conditions in electrodynamics was proposed over a century ago as a field-free variational action principle with direct action at a distance [50,51,52]. Dirac [14] introduced a theory of radiation-reaction in which boundary conditions in the future were used. This approach was formalized by Wheeler and Feynman, who called it absorber theory [15]. A quantum absorber theory was later developed [53], equivalent to quantum electrodynamics. The application of absorber theory to Bell violations [16,17] was used to explain the apparent lack of causality in Bell violations, which led to transactional quantum theory [54].

Criticisms were levelled at this approach [19,20], with the claim that retrocausality might lead to contradictions. In response, it was argued that such no-go theorems relied on untenable assumptions about probabilities in causal loops [23,55]. More recent reviews [56,57] have treated toy models of retrocausal quantum fields, and discuss these issues in more detail.

Objections have been raised to the assumption that realistic models must not be contextual, if “*one wishes some aspect of “reality” to be describable in a manner that is independent of the act of experimentation*” [58]. This raises the question of why one should wish this, since this assumption eliminates potential models including OQFT (unless we claim that the “aspect” is reality at a macroscopic level). Given that the *“act of experimentation”* is part of the universe, it is not clear why reality has to be independent of it.

We will discuss how one might resolve these questions using the objective model of real fields described in the previous section. In this model, the fields are regarded as having an objective existence, with retrocausal dynamics. We now outline the proof [9,11,28] of equivalence to the standard model of quantum field theory. The time evolution of quantum fields in quantum field theory (QFT) is conventionally described by the Dirac-Feynman quantum action principle, which has an imaginary exponent. This is equivalent to the Schrödinger equation,

(5) $$\frac{d\hat{\rho }\left(t\right)}{dt}=\frac{i}{\hbar }[\hat{\rho }\left(t\right),\hat{H}].$$

Equations (1) and (5) make it seem that P(ϕ,t) relies on a wave-function or density matrix $\hat{\rho }\left(t\right)$ evolving in time. However, this is not required. By using appropriate identities, one can obtain a partial differential equation, in the fields alone, of form

(6) $$\frac{dP(\phi ,t)}{dt}=DP\left(\phi ,t\right).$$

If the quantum Hamiltonian $\hat{H}$ has nonlinearities that are at most quartic in the quantum fields, D has up to second order partial derivatives in the fields ϕ. This is an algebraic property of the differential identities used to transform Equation (5) into the generalized Fokker–Planck Equation (6).

The resulting partial functional differential equation is similar to a Fokker–Planck equation, describing diffusion in a noisy environment, except that the diffusion matrix is not positive-definite, and has a zero trace. As a result, the trajectories obey a real action principle [59,60,61] of an unusual type [9,11]. The probability functional of a complete space-time trajectory $P\left[\phi \right]$ is expressed in terms of a time-symmetric Lagrangian $L_{\phi }\left(\phi ,\dot{\phi }\right)$, different to the conventional Lagrangian of mechanics. We suppress the space-dependence of fields in the following, for brevity. The path integral satisfies mixed (Robin-type) boundary conditions in the past and future:

(7) $$P\left[\phi \right]=\exp-\int L_{\phi }\left(\phi \left(t\right),\dot{\phi }\left(t\right)\right)dt\;.$$

In summary, time-evolution of the objective fields is described by an action principle which determines the probability of a particular trajectory $\phi \left(r\right)$ occurring, given boundary conditions in the past and the future. The probability of any particular evolution is defined according to a path integral. Unlike the Dirac–Feynman quantum action principle, the action is real. It has mixed boundary conditions both in the past and in the future. These fields are therefore neither the elements of reality of Einstein, Podolsky and Rosen [4], nor hidden variables in the sense of Bell [62].

Unlike the Dirac–Feynman path integral, the time-symmetric stochastic path integral cannot be used to quantize all interactions. If the Hamiltonian has higher than quartic nonlinearities, Equation (6) has third- or higher-order differential terms. In such cases, we know of no equivalent path-integral result. In the similar case of a forward Fokker–Planck equation, it is known that such equations have no probabilistic interpretation, except in certain infinite-order cases [63].

In the OQFT approach, interacting fields are divided into complementary quadratures

(8) $$\phi =\left[\phi^{+},\phi^{-}\right],$$

where there are two quadratures $\phi^{\pm }$. One propagates forward in time, one backward in time, so that the result of applying the action principle is a forward-backward stochastic differential equation, of the general form:

(9) $$\begin{array}{cc} \phi^{+}\left(t\right) & =\Phi^{+}\left(\phi^{-}\left(0\right)\right)+\int_{0}^{t}A^{+}\left[\phi \left(t^{\prime }\right)\right]dt^{\prime }+\int_{0}^{t}dw^{+}\left(t^{\prime }\right)\\ \phi^{-}\left(t\right) & =\Phi^{-}\left(\phi^{+}\left(T\right)\right)-\int_{t}^{T}A^{-}\left[\phi \left(t^{\prime }\right)\right]dt^{\prime }-\int_{t}^{T}dw^{-}\left(t^{\prime }\right) \end{array}$$

Here, $w^{\pm }$ are stochastic fields, and $A^{\pm }$ represent classical-like drift functionals. From the initial and final boundary conditions $\Phi^{\pm }$, $\phi^{-}\left(t\right)$ may depend on $\phi^{+}\left(T\right)$, and/or $\phi^{+}\left(t\right)$ may depend on $\phi^{-}\left(0\right)$. This gives an explicit cyclic dependence between the fields, in addition to those present in the forward-backwards equations due to cross-coupling. The combination of retrocausality and cyclic dependency is the difference between an objective field theory and conventional causal quantum mechanics.

The entire field ϕ(t,x) is the basic element of reality here. This can be transformed by relativistic frame transformations, so it is meaningful in any frame of reference, and is a relativistic invariant. The value of ϕ at a fixed time is not a unique ontological object. This would obviously be frame-dependent, since it depends both on the chosen frame of reference, and on a particular choice of time coordinate. The full trajectory $\phi \left(t,x\right)$ does not have this limitation of frame-dependence.

We cannot impose a unique time coordinate on a physical system in an observer-independent way. One therefore should not compare the objective field theory ontology with hidden variable theories [62], which propose that reality is described by a set of variables defined at a single time-like surface. Equally importantly, hidden variables are assumed to be independent of any future events.

There are limitations to these methods. Objective field trajectories *only* exist for Hamiltonians at most quartic in the field operators, which includes quantum electrodynamics [64,65,66,67], quantum chromodynamics [68,69] and the quartic Higgs-Englert-Guralnik model [29,30,31]: the standard model of physics. This covers all that is empirically known in quantum theory. The OQFT approach is able to treat the part of quantum field theory that is physically relevant. Higher-order nonlinearities do not have an OQFT description, and are not renormalisable. They are not considered fundamental in standard quantum field theory [70,71], although for different reasons.

By contrast, conventional Lagrangian methods [8] may include *any* nonlinear coupling, almost all of which are unphysical. Thus, OQFT is a more restricted theory than standard QFT. The limitation to quartic and cubic nonlinearities means that the OQFT approach is not applicable to most first quantized theories, which often use non-polynomial interaction potentials. An example is the Coulomb interaction. This is not a serious problem, because first quantization is not a fully correct physical theory.

Using more limited techniques might seem unproductive. We argue that the opposite is true. The “consensus” theory of epicycles in geocentric Ptolemaic astronomy was very flexible, but treated the earth as the center of the universe. The Copernican approach of Newton, although restricted, was more scientifically useful [72], and eliminated the earth-bound observer as being central. The present approach also removes an anthropomorphic bias: the Copenhagen observer. If OQFT is more restricted, it has the merit of being more easily falsified, and therefore complies with what is needed in a scientific theory [73].

Quantization of gravity is outside the scope of this article. Previous attempts at quantizing gravity are problematic. One issue is a lack of renormalisability [71,74], with infinities that cannot be removed. Another issue is how to treat time [75]. Covariance under frame transformations is essential in general relativity. This is inconsistent with the global role played by time in conventional quantum measurement theory. Such problems appear absent in OQFT, although further investigation is needed.

## 4. The Einstein-Podolsky-Rosen (EPR) Paradox

Bohr and Einstein were pioneers in the earliest years of quantum theory, and engaged in several famous debates [76]. It is often claimed that Bohr won these debates. Certainly the Copenhagen school that Bohr founded was very influential. Yet, Einstein, in his 1935 EPR paper, was also important in pointing out the subtle paradoxes that resulted from this approach and questioning the completeness of the wave-function description [4,77]. These issues are still debated today. Their argument led to a draw. Both Einstein and Bohr were correct in their own way.

It is straightforward to summarize Einstein’s views. His main conceptual guidelines are described in his autobiographical notes [78]. Diecks [79] has summarized Einstein’s essential principles for a physical theory as follows:

1. *“The theory should be objective, in the sense of observer independent;*
2. *The description should be about fields and/or particles located in space and time;*
3. *Everything that exists should have its counterpart in the theoretical description”.*

In work on the possible incompleteness of quantum mechanics, Einstein also introduced classically causal “elements of physical reality”, which are not thought to exist today [4].

Bohr’s rhetoric is sometimes criticized, but it rewards careful reading. His main philosophy [36] was that *“this (quantum) postulate implies a renunciation as regards the causal space-time co-ordination of atomic processes*”, and “*any observation of atomic phenomena will involve an interaction with the agency of observation not to be neglected*”. His riposte [37] to Einstein’s claim about the incompleteness of quantum theory repeated his point that the operational procedures of measurement are a vital part of any correct description of physical reality. This is surely not that unreasonable. To test a theory, one must do experiments, using instruments that are also part of any external objective reality. It should not be very surprising if reality is affected by these measurements.

This is not inconsistent with Einstein’s three principles enumerated above. The fact that a measuring device can disturb what it measures does not contradict the idea that there is objective behaviour in the universe, nor does this require an observer. Bohr summarized this in his response to the Einstein, Podolsky and Rosen paper [37]: “*In fact this new feature of natural philosophy means a radical revision of our attitude as regards physical reality*.”

We agree both with the need for treating the meter, and with a renunciation of traditional space-time causality. Similarly, the need for invariance under a change in reference frame was vital to Einstein’s relativity theory and also must be incorporated in any modern physical theory.

Bohr’s general statement was later summarized by others as implying a need to collapse the wave-function when describing measurements [8]. This extension of Bohr’s ideas was not proposed by Bohr himself. It is useful in obtaining predictions, but conceptually it is the main drawback of the approach. It is unclear how to define a measurement in the first place, and collapse is attributed by some to a type of mental process [80]. This makes it questionable that a wave-function is physically real.

Einstein’s third condition is often ignored, yet it is worth examination. When a measurement takes place, we are familiar with the idea that a system in an eigenstate yields an eigenvalue, which is regarded as existing beforehand, and is present in the theory. More typically, the measurement yields a random value which *cannot* be obtained from the wave-function alone. This does not satisfy Einstein’s criteria. Essentially, Born excused this failing by calling quantum mechanics a statistical theory, but he also recognized that it left an open question [13]: a statistical theory of *what?*

This open question is what the objective field approach attempts to answer.

Between them, Bohm and Bell took up the challenge of Einstein and Bohr, each trying to establish their understanding of “what is”. They were less adversarial than Einstein and Bohr, however. Bohm, extending earlier ideas of de Broglie, created a model of reality [25] to disprove a claim by von Neumann that realistic models were not possible [81,82]. The claim of von Neumann was only valid in regard to a limited class of models. Bohm’s theory described non-relativistic particles moving in a potential created by the non-relativistic Schrödinger wave-function, which is outside this class of theories. Yet it hardly seems to fit into a modern picture of quantum fields. This has been recognized, and relativistic extensions proposed, but they do not yet appear to be fully equivalent to quantum field theory in all respects [83].

The objective model described by Bohm does have drawbacks. It is non-local, with action at a distance between the particles. It also relies on solving for the wave-function evolution. On top of this, it adds real particles as a superstructure. This seems to lack a coherent picture, as one cannot easily obtain a simple, unified description. However, this theory does make it clear that a realistic model of quantum mechanics is not impossible, an important breakthrough given von Neuman’s earlier claim.

Bell’s contribution was to demonstrate which models cannot work [62], and to investigate how to introduce Lorenz invariant, objective models [84]. He analysed a class of theories he termed “local hidden variable theories”. These are a formalization taken from Einstein, Podolsky and Rosen’s work on the incompleteness of quantum mechanics. Such theories are not the same as Bohm’s, which were inherently non-local. Bell was interested in understanding what could be achieved with local theories, where there is no action at a distance. In doing so, he introduced an additional, apparently almost trivial assumption, that causality proceeds from the past to the future. In other words, Bell implied that one can always compute the future from the past*,* which is also inherent in classical mechanics, ignoring computability issues.

It is this important assumption that is discarded in the objective quantum field model. Given that all physical laws appear to be time-reversible, Bell’s assumption formalizes an anthropomorphic prejudice that appears inconsistent with objective reality. Without this assumption, quantum features can co-exist with an objective physical reality. One should not think that this gives one a license to change the past, as in a science fiction novel. The issue of time’s arrow is a much more macroscopic one, closely linked to questions like the growth of entropy and the expansion of the universe.

## 5. Other Approaches to Quantum Ontology

In this section, we give an overview of some other approaches to quantum ontology. The purpose of this is not to give a comprehensive review. Instead, we are concerned with the more popular interpretations. Those that have similarities to ours in particular are treated. This allows us to focus on differences that we believe are significant. The field is developing rapidly, so not all alternative pictures can be included here.

One current picture of reality is mostly surprising in its complexity. Everett’s original idea of a relative state interpretation [85] did not treat objective reality per se. However, this has been later interpreted as implying that the wave-function describes all possible quantum universes, and that these all exist at once [24]. There have been many suggestions on how to identify what one of these universes comprises. One picture [86] is that decoherence allows a particular universe to be selected.

This is not accepted by all the experts in this field. Bell, one of the deepest thinkers on quantum foundations, was opposed to this idea [84]. He pointed out that decoherence does not lead to a transition to any one universe. Instead, it simply causes entanglement between a system and its environment.

A development of this general approach is the model of consistent histories [87,88,89]. This attempts to obtain probabilities of histories of the universe that have internal consistency. In order to do this, one must obtain a set of projection operators describing the history. This is similar in practice to the Copenhagen model, since measurement and collapse of the wave-function is related to a projection.

In this model, one has a class operator for a history, composed of projection operators at different times $t_{1},\ldots t_{n}$:

(10) $${\hat{C}}_{H_{i}}={\hat{P}}_{i,n_{i}}\cdots {\hat{P}}_{i,2}{\hat{P}}_{i,1}.$$

Central to the approach is consistency. For an initial density matrix $\hat{\rho }$, a set of histories is termed consistent if

(11) $$Tr\left[{\hat{C}}_{H_{i}}\hat{\rho }{\hat{C}}_{H_{j}}^{†}\right]=0.$$

Quoting Omnes [90], there are three differences with the Copenhagen approach:

1. *The logical equivalence between an empirical datum... and the result of a measurement, which is a quantum property, becomes clearer.*
2. *There are two apparently distinct notions of probability in the new approach. One is abstract and directed toward logic, whereas the other is empirical and expresses the randomness of measurements.*
3. *The main difference lies in the meaning of the reduction rule for “wave packet collapse”. In the new approach, the rule is valid but no specific effect on the measured object can be held responsible for it. Decoherence in the measuring device is enough.*

Yet, it is unclear how to interpret the probabilities that are obtained this way, nor the implication of the times $t_{1},\ldots t_{n}$. However they are obtained, the times $t_{j}$ require a special reference frame. A choice like this appears incompatible with special and general relativity. In addition, the procedure may result in a set of probabilities that are not consistent with the laws of probability theory. There are many variations on this approach. There is also an approach called a “modal” theory, with similar properties [91].

A general difficulty with the multiverse, consistent histories, and modal approaches is that there is no clear way to identify what reality is, for example by uniquely obtaining the projectors ${\hat{P}}_{i,n_{i}}$ [92]. One may try to link the time-scale and projections with decoherence theory. This is not a precise formulation of reality in Born’s sense. It leaves unanswered the question of what is happening between the measurements or projections. Nor does it have the completeness property (3) of Einstein, concerning actual results and the randomness of measurements.

Such approaches clearly do not describe the nature of the reality *between* the projection times, nor do they account for the origin of the random results that are found whenever a real physical measurement is made. Therefore, there are large differences between OQFT and consistent histories. In particular, OQFT has a unique definition of what is meant by reality. It uses projectors defined by the coherent group-theoretic spaces underlying the field commutators. It does not employ arbitrary orthogonal projectors, nor a sequence of operations at successive times.

There are previous attempts to introduce randomness into quantum mechanics. We briefly mention three of them, Nelson’s stochastic mechanics [93,94], Parisi and Wu’s stochastic quantization [95], and a recent phase-space based approach [96]. Nelson’s approach is conceptually related to ours, although originally just for a first quantized theory. While the idea was pioneering, Nelson was unsure if the stochastic mechanics approach could replicate all of quantum mechanics, let alone quantum field theory, and there are known issues [97,98] involving repeated measurement. These problems were subsequently re-analysed and a resolution proposed that required using a wave-function and projection approach [99], which one would argue therefore does not resolve the original measurement problem. The Parisi and Wu approach of stochastic quantization in Euclidean space was intended to solve gauge-fixing problems in QFT. Rotating the path integrals to Minkowski space is not straightforward, and may lead to negative probabilities [100]. The approach of Budiyono and Rohrlich [96] uses both stochastic trajectories and distributions, which is similar to the de Broglie-Bohm theory.

## 6. Summary

In conclusion, this article compares the quantum picture of reality in an objective quantum field theory (OQFT) with that of previous quantum physicists. We show that there are very close similarities with the original concepts held to be most important by Einstein and Bohr. However, there are substantial differences with more recent approaches. An OQFT has no need for multiple universes that exist simultaneously. Wave-functions collapsing due to measurement do not occur. It places no special importance on decoherence, nor on mental processes. New and previously unknown physics causing decoherence is not excluded, but also not required. The objective field approach appears empirically adequate to describe modern quantum field theory results. Many of the original philosophies of Einstein and Bohr are unified in this picture.
